# Health‐care costs among people who use methamphetamine in Australia

**DOI:** 10.1111/dar.14043

**Published:** 2025-03-20

**Authors:** Anh Dam Tran, Rory Chen, Grant Sara, Rebecca McKetin

**Affiliations:** ^1^ National Drug and Alcohol Research Centre UNSW Sydney Sydney Australia; ^2^ NHMRC Clinical Trials Centre, Faculty of Health and Medicine The University of Sydney Sydney Australia; ^3^ Centre for Healthy Brain Ageing UNSW Sydney Sydney Australia; ^4^ Northern Clinical School, Faculty of Medicine and Health The University of Sydney Sydney Australia; ^5^ Discipline of Psychiatry, Faculty of Medicine and Health UNSW Sydney Sydney Australia

**Keywords:** cost, detox, health services utilisation, methamphetamine, residential rehabilitation

## Abstract

**Introduction:**

This study aims to estimate the drug treatment costs and health services utilisation costs among people who used different treatment modalities to treat methamphetamine dependence.

**Methods:**

This is a sub‐study of a prospective observational cohort study MATES (*n* = 501), including recruitment from 15 residential rehabilitation services, 13 counselling services and 11 detoxification units in Sydney and Brisbane, Australia. Participants with methamphetamine dependence who had used methamphetamine in the 28 days before baseline assessment and completed 3‐ and 12‐month follow‐ups were included (*n* = 323). Unit costs per day/episode of each treatment type were taken from published literature. Self‐reported health services utilisation (HSU) was collected via structured face‐to‐face and telephone interviews. Hospital admissions were allocated a likely diagnosis grouping from the Australian Refined Diagnosis‐Related Groupings. Emergency department presentations were allocated a likely triage category.

**Results:**

Average drug treatment costs per person per year were AU$10,540 (SD 13,995), accounting for 63% of total health‐care costs, which were AU$16,862 (SD 19,908). Drug treatment costs of counselling participants were only around 40% of detox and residential rehab participants. However, the HSU costs of detox and rehab participants were approximately 60% lower than those of counselling participants.

**Discussion and Conclusions:**

Drug treatment costs accounted for more than half of total health care costs, representing a substantial financial burden. Detox and rehab treatment costs more but saved costs in health services use. HSU costs among people who had severe mental health problems and injected methamphetamine were substantially high. Strategies to support these groups are necessary to reduce the cost burden.

**Key Points**

1. Average drug treatment costs were estimated at AU$ 10,540 per person per year.
2. Detoxification and residential rehabilitation had higher drug treatment costs but lower health services utilisation costs compared to counselling.
3. Health services use costs among people who had severe mental illness and injected methamphetamine use was substantially high.

## 1 INTRODUCTION

Countries worldwide are currently experiencing increases in methamphetamine use. In US adolescents and adults, the 12‐month prevalence of amphetamine use or amphetamine use disorder increased by more than 40% between 2016 and 2019 [1]. In Australians who inject drugs at least weekly, the proportion reporting methamphetamine use increased from 29% in 2011 to 51% in 2019 [2]. In Canadians aged 15 years and older, the lifetime prevalence of amphetamine use increased from 3.0% in 2013 to 3.7% in 2017 [3].

Methamphetamine use is associated with a range of health harms, including cardiovascular and renal dysfunction, psychosis, depression, sexually transmitted infections, overdose and increased mortality [4, 5, 6, 7, 8, 9]. In the United States, drug overdose death rates involving psychostimulants increased more than 300%, from 1.2 per 100,000 in 2013 to 5.0 per 100,000 in 2019 [10]. In Australia, the observed rate of amphetamine‐induced deaths per 100,000 persons increased from 0.14 in 2012 to 0.40 in 2018 [11]. In British Columbia, Canada, methamphetamines were detected in 33% of illicit drug overdose deaths between 2016 and 2019, increasing from 8% in 2008 and 29% in 2017 [12, 13].

Societal costs of methamphetamine use are necessary to justify resource allocation, inform policy and plan health‐care services. In Australia, to date, national societal costs of methamphetamine use were estimated at AU$3,731 million in 2004 [14] (including crime, health and traffic accidents) and AU$5023.8 million in 2013/2014 (including crime, premature mortality, health, workplace, child protection, traffic accident, harm reduction and clandestine laboratories) [15]. Health‐care costs were estimated at AU$341 million in 2004 by Moore et al. [14] and AU$270.8 million in 2013/2014 by Tait et al. [15]. Due to the unavailability of methamphetamine‐related mortality and morbidity data, both studies used attributable factors to estimate the health‐care costs.

In Australia, there are three common treatment modalities to treat methamphetamine dependence, including residential rehabilitation, detoxification and counselling [16]. Counselling consists of various therapeutic approaches (e.g., motivational speeches, cognitive behaviour therapy and mindfulness), with this treatment being tailored to the participant's needs and goals [17]. Detoxification is another form of treatment that aims to manage intense withdrawal symptoms from substance use and prevent dangerous adverse events. Detoxification can be delivered from a variety of settings, including home‐based or an inpatient facility [34]. Residential treatment is provided in live‐in facilities with 24‐h supervision for patients with significant substance abuse problems, who cannot deal with their addiction but do not meet the clinical criteria for hospitalisation [18]. Residential rehabilitation services around Australia usually offer different types of treatment, such as group psychotherapy and practical and vocational activities, coupled with individual case management [19].

In Australia, drug treatment costs per methamphetamine user have yet to be investigated. Treatment drop‐out, which is common among methamphetamine users [20, 21] may hinder the accurate estimation of drug treatment costs. The current study estimated the total drug treatment costs per methamphetamine user per year by including both complete and incomplete treatments over a year.

Specifically, we aim to: (i) estimate drug treatment costs, health services utilisation (HSU) costs per participant per year by treatment modality; (ii) examine the impact of treatment modality on drug treatment costs and HSU costs; (iii) pair‐wise compare drug treatment costs and HSU costs between treatment modalities; (iv) examine the association between participants' characteristics and HSU costs.

This is a sub‐study of a prospective observational cohort study MATES (*n* = 501), which included recruitment from from 15 residential rehabilitation services, 15 counselling services and 11 detoxification units in Sydney (*n* = 25) and Brisbane (*n* = 15), Australia.

## 2 METHODS

### 2.1 Overview of the study

In this sub‐study, participants with methamphetamine dependence who had used methamphetamine in the past 28 days at baseline and completed both 3‐ and 12‐month follow‐ups were included (*N* = 324). Data on health service use were available from 323 of these participants. All the costs were calculated based on the Government's perspective. Ethics approval was provided by the University of New South Wales and endorsed by all participating institutions. Details of the study have been published elsewhere [22]. This costing analysis was an post hoc analysis and was not pre‐registered.

### 2.2 Measurements

#### 2.2.1 Dependent variables—Drug treatment costs

Drug treatment costs (detoxification/counselling/residential rehabilitation) and HSU costs (inpatient/outpatient) in the past 12 months were used as the dependent variable. For each episode, participants were asked to provide treatment type, length and status (completed or not). Unit costs per day episode of each treatment type were taken from published literature.

Drug treatment costs were computed by summing the costs of each reported treatment episode in the last 12 months (including the episode at baseline, and episodes since the last interview at 3‐ and 12‐month follow‐up). For each episode, participants were asked to provide treatment type, length and status (completed or not) (Data S10). Unit costs per day episode of each treatment type were taken from published literature. Pharmacotherapy (methadone, buprenorphine) was not included in drug treatment costs. We did not include pharmacotherapy in our drug treatment cost because participants were supposed to complete pharmacotherapy before being accepted into residential rehab, so no pharmacotherapy was provided in residential rehab.

In Australia, the availability of drug treatment costs by treatment modality, for example, costs per day and cost per episode was very limited due to varied operation systems. Most available reported drug treatment costs were not specific to methamphetamine. Hence, the unit costs used in our estimation were the unit costs for all drugs of dependence.

For each treatment modality, if the treatment length was not provided, the costs were replaced with unit costs for a completed episode (if the participant ticked the option completed) and 50% of the unit costs for an uncompleted episode (if the participant ticked the option uncompleted). For some other treatment types outside of the three main modalities (counselling/detoxification/residential rehabilitation), we had face‐to‐face team meetings to discuss the nature of the treatment and reached a consensus about the treatment types and decided on the costs by searching their published unit costs.

#### 2.2.2 Dependent variables—Health services utilisation costs

HSU was collected at 1‐year follow‐up using a bespoke questionnaire (see Data S11). The questionnaire for collecting HSU was presented in Data S11. The questionnaire asked participants to describe several key service types in the preceding 1‐year period. Participants were also asked to identify episodes of care with general hospitals, emergency departments (ED), ambulances, psychiatric hospitals, psychiatrist visits, psychologists or counsellors, dentists and general practitioners. If the participants provided the length of the services, we used the unit costs per day times the length of stay. For some reported reasons that were not informative, we used average costs published in the Australian Institute of Health and Welfare report. We used New South Wales data if available because most of our participants were in New South Wales, if not, we used national data.

For each ED visit and general hospital admission, participants were asked to identify the primary reason for presentation or hospitalisation and the number of days spent in ED or hospital. The study did not have access to linked health‐care data. Three authors (Anh Dam Tran, Grant Sara, Rebecca McKetin) reviewed all records to estimate the clinical complexity of ED presentations and hospital admissions. ED presentations were allocated a likely triage category. Hospital admissions were allocated a likely diagnosis grouping from the Australian Refined Diagnosis‐Related Groupings (AR‐DRG). Levels of complexity and weights were assigned based on reasons for admission and length of stay, which were provided in the case report forms. We (Anh Dam Tran, Rebecca McKetin, Grant Sara) worked on each admission manually and linked different pages (mental health, drug use, ambulance, hospital admission) to understand the health condition of each participant before assigning the code and the weight. We assigned the AR‐DRG independently and conducted meetings (*n* = 5) to discuss until we reached a consensus. We also consulted staff in the finance department at Royal Prince Alfred Hospital for confirmation. Costs for all service types were estimated using published benchmark costs available for ED and hospital admission.

Author Grant Sara had expertise in hospital and emergency department data. Any conflicts were further discussed and solved with finance staff at the Ministry of Health. Based on AR‐DRG and length of stay, triage, cost weights for hospitalisations and emergency admissions were then extracted from reference [23]. Unit costs were then calculated by timing cost weights with the National Weighted Activity Unit [23]. We did not include medication costs because of the low quality of self‐reported data on medications.

#### 2.2.3 Independent variables—Treatment modality

The treatment modality in our study was the treatment the participant spent most of their time on over a year (excluding the index treatment). This variable was created by our validation based on the information we collected from self‐reported data. Therefore, the reported drug treatment costs in the residential rehabilitation group did not only include residential rehabilitation treatment but also all the treatments incurred over a year for that group (may also include detoxification or counselling treatment).

The predicted costs in Table 3 provided an estimate of the adjusted absolute costs for each treatment modality, assuming the average characteristics (or reference categories) of the study population. Adjusting for covariates in this way allowed us to compare the adjusted absolute costs of the different treatment modalities, controlling for the influence of other variables. This allowed us to provide a more intuitive comparison of absolute costs across treatment modalities while ensuring that the results were not biased by differences in the distribution of covariates across modalities. Therefore, the predicted costs could be interpreted as the expected costs for each treatment modality for a ‘typical’ individual with average characteristics. The ‘actual costs’ by modalities were reported in Table 2.

#### 2.2.4 Covariates

The analysis included a range of demographic, socio‐economic, illicit drug use history and mental and general health. Demographic and socio‐economic characteristics comprised age, gender, education, employment, marital status, accommodation type and history of incarceration. Illicit drug use history included ever‐injected drugs and the age of first methamphetamine use. Methamphetamine use in the month before treatment was captured through the frequency of use, severity of dependence scale scores, the way (injection or others) and the form (crystal or others) of methamphetamine used, and polydrug use. Mental and general health factors were also considered, including depression, panic disorder or social phobia, K10 Psychological Distress Scale, Jablensky Psychosis Screener and the SF‐12 Mental and Physical component, with the hypothesis that participants with mental illness may incur more health services utilisation costs.

### 2.3 Data analysis

Characteristics of participants were reported by treatment modality using Pearson's chi‐square tests or non‐parametric Kruskal–Wallis tests. All variables were categorised for reporting purposes (Table 1). The main outcome measures were drug treatment costs and HSU costs in the past 12 months. The main predictor variable was treatment modality. As all costs were non‐normally distributed (Shapiro–Wilk test, *p* < 0.05), descriptive analyses were conducted using the non‐parametric Kruskal–Wallis tests.

**TABLE 1:** Characteristics of participants by treatment modality.

	Included	Not in treatment	Detox	Counselling	Residential rehabilitation	*p*
N	324	70	59	29	166	
Demographics						
Age, median (IQR)	31 (25–37)	33 (30–40)	32 (27–38)	28 (20–35)	30 (25–35)	<0.01
Gender						0.15
Female	95 (29)	24 (34)	19 (32)	12 (41)	40 (24)	
Male	229 (71)	46 (66)	40 (68)	17 (59)	126 (76)	
Education in years, median (IQR)	10 (9–11)	10 (8–11)	10 (9–10)	10 (9–12)	10 (9–12)	0.22
Employment status						<0.01
Unemployed	259 (80)	61 (87)	40 (68)	12 (41)	146 (88)	
Others	65 (20)	9 (13)	19 (32)	17 (59)	20 (12)	
Fortnight legitimate income after‐tax						0.10
<$400	80 (25)	12 (17)	21 (36)	6 (21)	41 (25)	
≥$400	244 (75)	58 (83)	38 (64)	23 (79)	125 (75)	
Marital status						0.13
Others	263 (81)	54 (77)	47 (80)	20 (69)	142 (86)	
Married/de‐facto	61 (19)	16 (23)	12 (20)	9 (31)	24 (14)	
Accommodation						<0.01
Stable accommodation	202 (62)	26 (37)	36 (61)	22 (76)	118 (71)	
Public housing	48 (15)	23 (33)	6 (10)	3 (10)	16 (10)	
Other accommodation	74 (23)	21 (30)	17 (29)	4 (14)	32 (19)	
Prison history						<0.01
No	203 (63)	28 (40)	40 (68)	24 (83)	111 (67)	
Yes	121 (37)	42 (60)	19 (32)	5 (17)	55 (33)	
Drug use history						
Ever injected a drug						<0.01
No	46 (14)	4 (6)	9 (15)	10 (34)	23 (14)	
Yes	278 (86)	66 (94)	50 (85)	19 (66)	143 (86)	
Age first used methamphetamine, median (IQR)	17 (16–19)	17 (16–21)	17 (15–20)	16 (16–18)	17 (15–19)	0.40
Drug use in the month before treatment						
Number of days used methamphetamine, median (IQR)	16 (8–21)	12 (6–20)	20 (14–28)	15 (6–17)	16 (8–24)	<0.01
Methamphetamine Severity of Dependence Scale, median (IQR)	9 (6–12)	8 (5–10)	10 (8–12)	10 (8–11)	10 (7–12)	0.02
The main way to take methamphetamine						0.06
Others	82 (25)	10 (14)	15 (25)	11 (38)	46 (28)	
Injection	242 (75)	60 (86)	44 (75)	18 (62)	120 (72)	
The main form of methamphetamine						0.18
Others	139 (43)	24 (35)	31 (53)	15 (52)	69 (42)	
Crystal meth or ice	183 (57)	44 (65)	28 (47)	14 (48)	97 (58)	
Polydrug use in the past 28 days, median (IQR)	4 (3–5)	4 (3–5)	3 (3–4)	3 (2–4)	4 (3–5)	0.02
Mental and general health						
Major depression						<0.01
No	172 (53)	25 (36)	38 (64)	16 (55)	93 (56)	
Yes	152 (47)	45 (64)	21 (36)	13 (45)	73 (44)	
Panic disorder or social phobia						0.01
No	203 (63)	37 (53)	47 (80)	19 (66)	100 (60)	
Yes	121 (37)	33 (47)	12 (20)	10 (34)	66 (40)	
K10 Psychological Distress Scale	32 (27–37)	29 (22–36)	32 (26–39)	29 (26–36)	34 (29–38)	<0.01
Jablensky Psychosis Screener						0.33
No	78 (24)	20 (29)	16 (27)	9 (31)	33 (20)	
Yes	246 (76)	50 (71)	43 (73)	20 (69)	133 (80)	
SF12 mental health component, median (IQR)	27 (22–36)	32 (26–43)	28 (21–38)	29 (23–34)	25 (21–32)	<0.01
SF12 physical health component, median (IQR)	47 (40–55)	44 (39–55)	50 (39–55)	48 (41–53)	47 (40–56)	0.54

Abbreviation: IQR, interquartile range.

Due to the highly skewed distribution of costs, mean and standard deviation, median and interquartile range were both reported. In addition to the generalised linear model (glm) with a gamma distribution, post‐hoc Tukey–Kramer pairwise comparisons were conducted to examine the cost differences between treatment modalities controlling for participants' characteristics [24]. Glm is a mathematical framework for expressing relationships among variables that can express linear relationships between a numerical dependent variable and any combination of categorical or continuous independent variables. The gamma distribution was selected based on the lowest value of the Akaike information criterion and the Bayesian information criterion. Data were analysed using SAS 9.4. All statistical tests were performed at the 0.05 level of significance and were two‐sided.

## 3 RESULTS

### 3.1 Characteristics of participants

Among 324 participants, 71% were male, 80% were unemployed, 81% were not married, 86% had injected drugs and 64% had severe mental disorders (K10 score >30), 76% answered yes to the Jablensky Psychosis Screener, and 82% had SF‐12 mental ≥40. Characteristics that differed between treatment modalities were presented in Table 1.

### 3.2 Drug treatment costs and HSU costs per participant per year, by treatment modality

Average total health care costs per person per year among 323 participants who used methamphetamine in our study were AU$16,862 (SD 19,908), of which 63% was drug treatment costs (AU$10,540 [SD 13,995]), and 37% was from health services utilisation (AU$6316 [SD: 12,336]) (Table 2).

**TABLE 2:** Unadjusted drug treatment costs, health services utilisation (HSU) costs and total health‐care costs per participant per year, by treatment modality.

	*n*	Mean	SD	Median	Q1–Q3
Drug treatment costs					
Not in treatment	70	1499	4077	0	0–0
Detoxification	59	15,268	21,518	6720	3360–19,320
Counselling	29	5067	5951	2259	2140–5020
Residential rehabilitation	166	13,628	12,253	8909	6786–16,935
Total	324	10,540	13,995	7267	2182–12,663
HSU costs					
Not in treatment	70	5100	6503	2229	579–6448
Detoxification	59	5245	8134	1147	448–5353
Counselling	29	13,632	24,830	1492	552–16,895
Residential rehabilitation	165	5929	11,698	1524	552–4084
Total	323	6316	12,236	1643	550–5117
Total health‐care costs					
Not in treatment	70	6599	7677	2855	658–10,703
Detoxification	59	20,513	23,803	11,392	5488–25,914
Counselling	29	18,699	26,660	5474	3446–22,776
Residential rehabilitation	165	19,587	19,334	11,793	8215–26,338
Total	323	16,862	19,908	9941	4507–21,858

### 3.3 Impact of treatment modality on drug treatment cost and HSU costs adjusted for covariates

Participants who spent the most time in detox and residential rehabilitation had the highest drug treatment costs (AU$13,091 and AU$12,463, respectively). Participants who spent the most time in counselling services had the highest HSU costs (AU$9327) (Table 3).

**TABLE 3:** Impact of treatment modality on drug treatment cost, HSU and total cost, adjusted for participants' characteristics.

	Estimate	SE	Lower	Upper	*p*‐value	Estimated costs (95% CI)
Drug treatment costs						
Counselling	−0.17	0.29	−0.74	0.41	0.56	5075 (3513–7331)
Detoxification	0.78	0.27	0.26	1.30	**<0.01**	13,091 (9548–17,947)
Residential rehabilitation	0.73	0.25	0.24	1.21	**<0.01**	12,463 (9276–16,744)
Not in treatment	–	–	–	–		6011 (3634–9942)
HSU costs						
Counselling	0.88	0.324	0.25	1.52	**0.01**	9327 (5581–15,588)
Detoxification	0.03	0.29	−0.53	0.59	0.91	3988 (2423–6563)
Residential rehabilitation	−0.06	0.22	−0.50	0.38	0.79	3633 (2324–5681)
Not in treatment	–	–	–	–		3860 (2493–5975)
Total health‐care costs						
Counselling	0.99	0.23	0.54	1.44	**<0.01**	16,245 (11,234–23,491)
Detoxification	1.24	0.19	0.86	1.62	**<0.01**	20,886 (15,161–28,772)
Residential rehabilitation	1.09	0.16	0.78	1.40	**<0.01**	18,022 (13,318–24,389)
Not in treatment	–	–	–	–		6041 (4394–8306)

*Note*: All adjusting participant characteristics are presented in Table S4. The estimated costs in the last column were calculated taking into account all adjusting variables presented in Table S4.

Abbreviations: CI, confidence interval; HSU, health services utilisation.

### 3.4 Pair‐wise comparing drug treatment costs, HSU costs between treatment modalities

After adjusting for covariates, drug treatment costs of people who spent most time in counselling services were approximately two‐fifths (2/5) of detox and residential rehabilitation groups (Table 5). However, HSU costs among the counselling group were approximately three times those of the detox and residential rehabilitation groups, making the total health‐care costs of the counselling group not significantly higher compared to the detox or residential rehabilitation group (Table 4).

**TABLE 4:** Pairwise two‐sided multiple comparison analysis with Tukey‐Kramer adjustment.

	Drug treatment costs			HSU costs			Total health‐care costs		
	Detoxification	Residential rehabilitation	Not in treatment	Detoxification	Residential rehabilitation	Not in treatment	Detoxification	Residential rehabilitation	Not in treatment
Residential rehabilitation		–	**2.07 (1.28–3.37)**			0.96 (0.62–1.49)			**2.92 (2.15–3.96)**
Detoxification		1.05 (0.79–1.4)	**2.18 (1.29–3.67)**		1.05 (0.67–1.65)	1.01 (0.57–1.77)		1.14 (0.85–1.54)	**3.34 (2.30–4.85)**
Counselling	**0.39 (0.25–0.59)**	**0.41 (0.27–0.61)**	0.84 (0.48–1.5)	**2.55 (1.28–5.09)**	**2.68 (1.43–5.03)**	**2.57 (1.36–4.83)**	0.81 (0.51–1.29)	0.93 (0.60–1.42)	**2.70 (1.72–4.24)**

Abbreviation: HSU, health services utilisation.

**TABLE 5:** Health services utilisation (HSU) costs by participant characteristics.

	HSU costs		
	Mean (SD)	Median (IQR)	*p*‐value**
Age			0.44
≤31	7856 (14,881)	1626 (552–7880)	
>31	4767 (8583)	1790 (507–4620)	
Gender			**<0.01**
Female	9539 (14,285)	3134 (895–15,702)	
Male	4973 (11,033)	1264 (448–3658)	
Education			0.98
Completed <10 years of school education	5604 (9227)	1792 (579–3807)	
Completed ≥10 years of school education	6622 (13,328)	1620 (477–5353)	
Employment status			0.60
Unemployed	6103 (11,924)	1791 (552–5354)	
Others	7165 (13,471)	1276 (403–4672)	
Fortnight legitimate income after‐tax			0.90
<$400	5964 (10,064)	2001 (407–4619)	
≥$400	6432 (12,888)	1610 (552–5353)	
Marital status			0.72
Others	6641 (13,125)	1775 (552–5353)	
Married/de‐facto	4920 (7193)	1492 (411–4058)	
Accommodation			0.59
Stable accommodation	6233 (12,766)	1620 (448–5353)	
Public housing	6665 (11,979)	1894 (846–5502)	
Other accommodation	6317 (10,995)	1335 (560–4678)	
Prison history			0.36
No	6990 (13,390)	1749 (550–6385)	
Yes	5192 (9970)	1624 (550–4084)	
Ever injected a drug			0.12
No	6334 (16,655)	1119 (448–2361)	
Yes	6313 (11,401)	1795 (550–5500)	
Number of days used methamphetamine in the 28 days before entered treatment/past 28 days			0.58
1–14 days	6214 (13,960)	1443 (556–4065)	
15+ days	6403 (10,602)	1951 (432–6933)	
Main way took methamphetamine in the 28 days before entering treatment/past 28 days			**0.04**
Others	5482 (14,269)	1133 (448–3081)	
Injection	6596 (11,495)	1876 (560–5858)	
The main form of methamphetamine used in the 28 days before entered treatment/past 28 days			0.79
Others	5182 (8302)	1690 (552–4567)	
Crystal meth or ice	7143 (14,506)	1643 (454–5577)	
Methamphetamine Severity of Dependence Scale			0.32
Low to moderate (0–9)	4894 (8457)	1459 (552–3996)	
High (10–15)	7839 (15,163)	2004 (448–6198)	
Polydrug use in the past 28 days			0.80
1–3 drugs	5814 (9016)	1492 (552–5117)	
4+ drugs	6722 (14,419)	1797 (507–5049)	
Major depression			**<0.01**
No	5395 (11,479)	1152 (407–3604)	
Yes	7366 (13,004)	2207 (858–7880)	
Panic disorder or social phobia			**<0.01**
No	4802 (9187)	1194 (411–4045)	
Yes	8844 (15,814)	2148 (1013–9261)	
K10 Psychological Distress scale			**0.01**
No or mild mental disorder (10–24)	4054 (7083)	1226 (432–3142)	
Moderate mental disorder (25–29)	5480 (14,563)	1169 (388–3501)	
Severe mental disorder (30+)	7201 (12,591)	2070 (723–7776)	
Jablensky psychosis screener			0.22
No	4783 (8773)	1193 (448–4783)	
Yes	6805 (13,126)	1792 (550–5354)	
SF12 mental health component scale score			**0.02**
Nil or mild (<40)	3336 (5618)	1074 (373–3996)	
Moderate or severe stability (≥40)	6955 (13,150)	1904 (552–5354)	
SF12 physical health component scale score			0.07
Nil or mild (<40)	6129 (12,276)	1335 (448–4672)	
Moderate or severe stability (≥40)	6849 (12,180)	2224 (895–6153)	

Abbreviation: IQR, interquartile range.

** *p*‐value is reported for the median.

### 3.5 HSU costs by participants' characteristics

Females (AU$9539) had significantly higher HSU costs than males (AU$4973). Participants who injected methamphetamine in the 28 days before entering treatment had significantly higher HSU costs (AU$6596 vs. AU$5482). Depressed participants had significantly higher HSU costs: AU$7366 for major depression, AU$8844 for panic disorder/social phobia, AU$7201 (for those whose K10 ≥ 30), and AU$6955 (for those whose SF‐12 mental health component scale score ≥40).

## 4 DISCUSSION

Drug treatment costs in our study were substantially high, accounting for 63% of total health‐care costs. We used the estimated costs per day for residential rehabilitation and average costs per episode for counselling and detoxification to estimate the costs in our study, which may have also overestimated the drug treatment costs, especially for residential rehabilitation when the costs of residential rehabilitation vary substantially among services. For some participants who dropped out of treatment or did not report the completed day, we assumed the drug treatment costs to be half of the costs they incurred in the full episode. This assumption may have overestimated the real costs.

The average health services utilisation costs in our study were AU$6316 (SD 12,236). Moore et al. estimated the health services utilisation costs of methamphetamine use (*n* = 73,257) at 274 million in 2003, equivalent to $3740 per person per year. The adjusted number equated to $6240 in the year 2019 [25] which was approximate to the health services utilisation costs in our study. Tait et al. estimated national health services utilisation costs (*n* = 160,000) at 270.8 million in 2013/2014. The health services utilisation costs per person would be $1693, equivalent to approximately $2231 in the year 2019, which was far less than our estimation. Making comparisons between different studies is not straightforward due to the highly skewed distribution of cost data, differences in the cost structures, prevalence of methamphetamine use, and causal assumptions. It should also be noted that our health services utilisation was primary data, whereas three studies [14, 15, 26] estimated costs from different sources.

Participants who spent most of their time in residential rehabilitation had lower HSU costs compared to those who spent most of their time in detoxification and counselling. This may be because participants attending residential rehabilitation have fewer health problems due to reduced use of methamphetamine [26, 27, 28, 29]. Residential rehabilitation participants in our sample showed large reductions in the frequency of methamphetamine use at 3 months (odds ratio [OR] = 0.23), 1 year (OR 0.62) and 3 years (OR = 0.71) [22].

In our study, those who injected methamphetamine and had mental health problems at baseline had significantly higher HSU costs. More frequent methamphetamine use was associated with more frequent presentations to emergency departments and psychiatric hospitals [30]. Mental health was also cited as the top medical condition associated with ED visits [31]. Our finding that females had lower drug treatment costs than males but higher HSU costs than males is counterintuitive, with two studies reporting males were associated with hospitalisations [31, 32]. Sample selection may likely have influenced the discrepancy.

Our study had several strengths. We estimated the drug treatment costs by costing all the treatments the participants had in a year. The health services utilisation was methamphetamine‐specific, and the costs were reported by both demographic and drug characteristics. As far as we are aware, these have never been done before in Australia. Our study had limitations. Costs estimated in our study were based on participants in Sydney and Brisbane only. Generalisation of our cost estimation to another setting should be done with caution. We estimated the cost by patient level; however, the data were based on self‐reports which were subject to recall bias [33]. Some participants did not report the entry and exit dates, so the length of the treatment could not be calculated. We had to assume that participants finished half of the episode. This could lead to under‐ or overestimation. We assigned Medicare Benefits Schedule and Australian Refined Diagnosis Related Group item numbers from reported reasons for admission, based on our clinical expertise (author Grant Sara), clinician opinions, and consulting finance staff. This is considered a “best estimate” and could also under‐ or over‐estimate the costs. Although there were not many cases, this limitation should also be noted. We did not ask the participants about the location of the outpatient clinics they visited. Hospital‐based outpatient clinics could have different unit costs compared to standalone outpatient clinics because they are covered by the Tier 2 non‐admitted patient code. This could lead to different assigned unit costs. For drug treatment costs, research on the costs of providing drug and alcohol treatment in Australia has primarily focused on treatments for heroin dependence, with little research examining the actual costs for methamphetamine. Therefore, estimates should be treated with caution because external generalisability has not been shown. We collected medication costs, but we did not include them in our study because of the low quality of data. Further, we used the best estimate from available sources. We did not use the low bound and high bound of estimates. We therefore did not conduct a sensitivity analysis with different estimates. Our study reported total health‐care costs among methamphetamine users; we did not report societal costs (criminal costs, traffic, employment, childcare or mortality costs) which could be substantial for methamphetamine users. Our study looked at absolute costs among people using methamphetamine, not marginal costs versus the general population.

## 5 CONCLUSION

Methamphetamine users have high health‐care costs. Detoxification and residential rehabilitation treatment could incur higher drug treatment costs but save health services utilisation costs than counselling. Health services utilisation costs among people who have severe mental health problems and inject methamphetamine are substantially high. Strategies to support these groups are necessary to reduce the cost burden.

## AUTHOR CONTRIBUTIONS

Anh Dam Tran: Designed the study, conducted a systematic review, collected costing data, calculated drug treatment costs, and health services utilisation costs, and wrote the manuscript. Rory Chen: Led complex data analysis involving calculating the number and type of treatment exposures, hospital admissions, and ED contacts, and wrote part of the manuscript. Grant Sara: Provided expertise on hospital and emergency department data. Rebecca McKetin: Chief Investigator of the study, designed the study, wrote part of the manuscript, supervised the team, and commented on the manuscript.

## CONFLICT OF INTEREST STATEMENT

None.

## Supporting information


**Data S1** Supporting information.
