# Supplementary material for: Health‐care costs among people who use methamphetamine in Australia
Source: Drug Alcohol Rev. 2025 Mar 20;44(4):1157–68. doi: 10.1111/dar.14043 (PMC12117294; doi:10.1111/dar.14043)
Supplement: Supplementary file 1 — Data S1 Supporting information. [file DAR-44-1157-s001.docx]

**Table S1. Demographic characteristics of included and not included participants**

|  | **Not included** | **Included** | **p-value** |
| --- | --- | --- | --- |
|  | N (%) | N (%) |  |
| **N** | 177 | 324 |  |
| **Treatment modality** | | | |
| Not in treatment | 31 (18) | 70 (22) | **0.02** |
| Detoxification | 53 (30) | 59 (18) |  |
| Counselling | 11 (6) | 29 (9) |  |
| Residential rehabilitation | 82 (46) | 166 (51) |  |
| **Demographics** | | | |
| Age, median (IQR) | 31 (26-36) | 31 (25-37) | 0.98 |
| Gender |  |  | 0.11 |
| Female | 40 (23) | 95 (29) |  |
| Male | 137 (77) | 229 (71) |  |
| Education in years, median (IQR) | 10 (9-11) | 10 (9-11) | **0.02** |
| Employment status |  |  | 0.24 |
| Unemployed | 149 (84) | 259 (80) |  |
| Others | 28 (16) | 65 (20) |  |
| Fortnight legitimate income after-tax |  |  | 0.12 |
| <$400 | 55 (31) | 80 (25) |  |
| ≥$400 | 122 (69) | 244 (75) |  |
| Marital status |  |  | 0.40 |
| Others | 149 (84) | 263 (81) |  |
| Married/de-facto | 28 (16) | 61 (19) |  |
| Accommodation |  |  | 0.69 |
| Stable accommodation | 104 (59) | 202 (62) |  |
| Public housing | 27 (15) | 48 (15) |  |
| Other accommodation | 46 (26) | 74 (23) |  |
| Prison history |  |  | **<0.01** |
| No | 82 (46) | 203 (63) |  |
| Yes | 95 (54) | 121 (37) |  |
| **Drug use history** | | | |
| Ever injected a drug |  |  | 0.75 |
| No | 27 (15) | 46 (14) |  |
| Yes | 150 (85) | 278 (86) |  |
| Age first used methamphetamine, median (IQR) | 17 (15-20) | 17(16-19) | 0.95 |
| **Drug use in the month before treatment** | | | |
| Number of days used methamphetamine, median (IQR) | 16 (8-24.5) | 16 (8-21) | 0.62 |
| Methamphetamine Severity of Dependence Scale, median (IQR) | 8 (6-11) | 9 (6-12) | **<0.01** |
| The main way to take methamphetamine |  |  | 0.60 |
| Others | 43 (28) | 82 (25) |  |
| Injection | 113 (72) | 242 (75) |  |
| The main form of methamphetamine |  |  | 0.23 |
| Others | 58 (37) | 139 (43) |  |
| Crystal meth or ice | 97 (63) | 183 (57) |  |
| Polydrug use in the past 28 days, median (IQR) | 3 (3-4) | 4 (3-5) | **0.07** |
| **Mental and general health** | | | |
| Major depression |  |  | 0.11 |
| No | 107 (60) | 172 (53) |  |
| Yes | 70 (40) | 152 (47) |  |
| Panic disorder or social phobia |  |  | 0.52 |
| No | 116 (66) | 203 (63) |  |
| Yes | 61 (34) | 121 (37) |  |
| K10 Psychological Distress Scale, median (IQR) | 30 (24-36) | 32 (27-37) | **0.02** |
| Jablensky Psychosis Screener |  |  | **0.03** |
| No | 59 (33) | 78 (24) |  |
| Yes | 118 (67) | 246 (76) |  |
| SF12 mental health component, median (IQR) | 30 (22-38) | 27 (22-36) | 0.14 |
| SF12 physical health component, median (IQR) | 46 (41-55) | 47 (40-55) | 0.79 |

IQR, interquartile range.

**Table S2. Associated factors of health services utilisation costs**

| **Parameters** |  | **Estimate** | **SE** | **Lower** | **Upper** | **p-value** |
| --- | --- | --- | --- | --- | --- | --- |
| Treatment modalities | Counselling | 0.8823 | 0.324 | 0.2473 | 1.5174 | **0.01** |
|  | Detoxification | 0.0327 | 0.2869 | -0.5296 | 0.5949 | 0.91 |
|  | Residential rehabilitation | -0.0604 | 0.2226 | -0.4967 | 0.3758 | 0.79 |
|  | Not in treatment | – | – | – | – |  |
| Age |  | -0.0361 | 0.0105 | -0.0567 | -0.0155 | **<0.01** |
| Gender | Female | 0.5655 | 0.1926 | 0.1879 | 0.943 | **<0.01** |
|  | Male | – | – | – | – |  |
| Education | Completed <10 years of school education | -0.253 | 0.1761 | -0.5982 | 0.0922 | 0.15 |
|  | Completed ≥10 years of school education | – | – | – | – |  |
| Employment status | Others | -0.0183 | 0.209 | -0.4279 | 0.3912 | 0.93 |
|  | Unemployed | – | – | – | – |  |
| Fortnight legitment income after-tax | <$400 | -0.1747 | 0.1816 | -0.5306 | 0.1811 | 0.34 |
|  | ≥$400 | – | – | – | – |  |
| Marital status | Married/de-facto | -0.4515 | 0.2056 | -0.8544 | -0.0486 | **0.03** |
|  | Others | – | – | – | – |  |
| Accommodation | Other accommodation | -0.0759 | 0.1888 | -0.446 | 0.2941 | 0.69 |
|  | Public housing | 0.0322 | 0.2436 | -0.4454 | 0.5097 | 0.89 |
|  | Stable accommodation | – | – | – | – |  |
| Prison history | No | 0.2178 | 0.167 | -0.1096 | 0.5452 | 0.19 |
|  | Yes | – | – | – | – |  |
| Ever injected a drug | No | 0.1441 | 0.3158 | -0.4748 | 0.7631 | 0.65 |
|  | Yes | – | – | – | – |  |
| Number of days used methamphetamine in the 28 days before entered treatment/past 28 days |  | 0.0261 | 0.011 | 0.0046 | 0.0476 | **0.02** |
| Main way took methamphetamine in the 28 days before entering treatment/past 28 days | Injection | 0.7264 | 0.2696 | 0.1979 | 1.2548 | **0.01** |
|  | Others | – | – | – | – |  |
| The main form of methamphetamine used in the 28 days before entered treatment/past 28 days | Crystal meth or ice | 0.2185 | 0.1676 | -0.1101 | 0.547 | 0.19 |
|  | Others | – | – | – | – |  |
| Methamphetamine Severity of Dependence Scale |  | 0.0506 | 0.0231 | 0.0053 | 0.096 | 0.03 |
| Polydrug use in the past 28 days |  | -0.0164 | 0.0569 | -0.128 | 0.0952 | 0.77 |
| Major depression | No | -0.1571 | 0.19 | -0.5295 | 0.2153 | 0.41 |
|  | Yes | – | – | – | – |  |
| Panic disorder or social phobia | No | -0.4615 | 0.1904 | -0.8346 | -0.0884 | **0.02** |
|  | Yes | – | – | – | – |  |
| K10 Psychological Distress scale |  | 0.0126 | 0.0164 | -0.0195 | 0.0448 | 0.44 |
| Jablensky psychosis screener | No | 0.1045 | 0.1908 | -0.2694 | 0.4785 | 0.58 |
|  | Yes | – | – | – | – |  |
| SF12 mental health component scale score |  | 0.0015 | 0.0113 | -0.0206 | 0.0237 | 0.89 |
| SF12 physical health component scale score |  | -0.0159 | 0.009 | -0.0334 | 0.0017 | 0.08 |

**Table S3. Associated factors of total healthcare costs**

| **Parameters** |  | **Estimate** | **SE** | **Lower** | **Upper** | **p-value** |
| --- | --- | --- | --- | --- | --- | --- |
| Treatment modalities | Counselling | 0.9892 | 0.2286 | 0.5412 | 1.4372 | **<0.01** |
|  | Detoxification | 1.2405 | 0.1924 | 0.8634 | 1.6176 | **<0.01** |
|  | Residential rehabilitation | 1.0930 | 0.1584 | 0.7825 | 1.4035 | **<0.01** |
|  | Not in treatment | – | – | – | – |  |
| Age |  | -0.0123 | 0.0076 | -0.0271 | 0.0025 | 0.10 |
| Gender | Female | 0.1234 | 0.1277 | -0.1268 | 0.3736 | 0.33 |
|  | Male | – | – | – | – |  |
| Education | Completed <10 years of school education | -0.0285 | 0.1211 | -0.2659 | 0.2089 | 0.81 |
|  | Completed ≥10 years of school education | – | – | – | – |  |
| Employment status | Others | 0.1211 | 0.1413 | -0.1559 | 0.3981 | 0.39 |
|  | Unemployed | – | – | – | – |  |
| Fortnight legitment income after-tax | <$400 | 0.046 | 0.1275 | -0.204 | 0.2959 | 0.72 |
|  | ≥$400 | – | – | – | – |  |
| Marital status | Married/de-facto | -0.0832 | 0.1382 | -0.3541 | 0.1877 | 0.55 |
|  | Others | – | – | – | – |  |
| Accommodation | Other accommodation | -0.1082 | 0.1317 | -0.3664 | 0.1499 | 0.41 |
|  | Public housing | 0.0023 | 0.1662 | -0.3234 | 0.328 | 0.99 |
|  | Stable accommodation | – | – | – | – |  |
| Prison history | No | 0.1401 | 0.1142 | -0.0838 | 0.3639 | 0.22 |
|  | Yes | – | – | – | – |  |
| Ever injected a drug | No | -0.0304 | 0.2197 | -0.461 | 0.4002 | 0.89 |
|  | Yes | – | – | – | – |  |
| Number of days used methamphetamine in the 28 days before entered treatment/past 28 days |  | 0.0031 | 0.0072 | -0.0109 | 0.0172 | 0.66 |
| Main way took methamphetamine in the 28 days before entering treatment/past 28 days | Injection | 0.3263 | 0.1811 | -0.0288 | 0.6813 | 0.07 |
|  | Others | – | – | – | – |  |
| The main form of methamphetamine used in the 28 days before entered treatment/past 28 days | Crystal meth or ice | 0.1482 | 0.1114 | -0.0702 | 0.3667 | 0.18 |
|  | Others | – | – | – | – |  |
| Methamphetamine Severity of Dependence Scale |  | 0.0385 | 0.0167 | 0.0058 | 0.0711 | **0.02** |
| Polydrug use in the past 28 days |  | 0.0169 | 0.0382 | -0.058 | 0.0918 | 0.66 |
| Major depression | No | -0.1089 | 0.1211 | -0.3461 | 0.1284 | 0.37 |
|  | Yes | – | – | – | – |  |
| Panic disorder or social phobia | No | -0.2715 | 0.123 | -0.5126 | -0.0304 | **0.03** |
|  | Yes | – | – | – | – |  |
| K10 Psychological Distress scale |  | 0.0122 | 0.0117 | -0.0107 | 0.0352 | 0.30 |
| Jablensky psychosis screener | No | 0.1393 | 0.131 | -0.1174 | 0.3959 | 0.29 |
|  | Yes | – | – | – | – |  |
| SF12 mental health component scale score |  | 0.0017 | 0.0075 | -0.0131 | 0.0164 | 0.83 |
| SF12 physical health component scale score |  | -0.0069 | 0.0063 | -0.0192 | 0.0054 | 0.27 |

**Table S4. Impact of treatment modality on drug treatment cost, health services utilisation (HSU) and total cost, adjusted for all covariates**

| **Parameters** |  | **Estimate** | **SE** | **Lower** | **Upper** | **p-value** |
| --- | --- | --- | --- | --- | --- | --- |
| **Drug treatment costs** | | | | | | |
| Treatment modalities | Counselling | -0.1692 | 0.2932 | -0.7438 | 0.4054 | 0.56 |
|  | Detoxification | 0.7783 | 0.2664 | 0.2562 | 1.3004 | **<0.01** |
|  | Residential rehabilitation | 0.7292 | 0.2477 | 0.2437 | 1.2147 | **<0.01** |
|  | Not in treatment | – | – | – | – |  |
| **HSU costs** | | | | | | |
| Treatment modalities | Counselling | 0.8823 | 0.324 | 0.2473 | 1.5174 | **0.01** |
|  | Detoxification | 0.0327 | 0.2869 | -0.5296 | 0.5949 | 0.91 |
|  | Residential rehabilitation | -0.0604 | 0.2226 | -0.4967 | 0.3758 | 0.79 |
|  | Not in treatment | – | – | – | – |  |
| **Total healthcare costs** | | | | | | |
| Treatment modalities | Counselling | 0.9892 | 0.2286 | 0.5412 | 1.4372 | **<0.01** |
|  | Detoxification | 1.2405 | 0.1924 | 0.8634 | 1.6176 | **<0.01** |
|  | Residential rehabilitation | 1.093 | 0.1584 | 0.7825 | 1.4035 | **<0.01** |
|  | Not in treatment | – | – | – | – |  |
| Age |  | 0.0048 | 0.0084 | -0.0118 | 0.0213 | 0.57 |
| Gender | Female | -0.1935 | 0.1379 | -0.4638 | 0.0767 | 0.16 |
|  | Male | – | – | – | – |  |
| Education | Completed <10 years of school education | 0.058 | 0.1274 | -0.1918 | 0.3077 | 0.65 |
|  | Completed ≥10 years of school education | – | – | – | – |  |
| Employment status | Others | 0.0178 | 0.1485 | -0.2733 | 0.3088 | 0.90 |
|  | Unemployed | – | – | – | – |  |
| Fortnight legitment income after-tax | <$400 | 0.1755 | 0.1309 | -0.0809 | 0.432 | 0.18 |
|  | ≥$400 | – | – | – | – |  |
| Marital status | Married/de-facto | 0.0757 | 0.1505 | -0.2193 | 0.3707 | 0.62 |
|  | Others | – | – | – | – |  |
| Accommodation | Other accommodation | -0.1387 | 0.1398 | -0.4126 | 0.1353 | 0.32 |
|  | Public housing | 0.1088 | 0.1864 | -0.2565 | 0.4741 | 0.56 |
|  | Stable accommodation | – | – | – | – |  |
| Prison history | No | 0.1044 | 0.1245 | -0.1395 | 0.3484 | 0.4 |
|  | Yes | – | – | – | – |  |
| Ever injected a drug | No | -0.089 | 0.2173 | -0.515 | 0.3369 | 0.68 |
|  | Yes | – | – | – | – |  |
| Number of days used methamphetamine in the 28 days before entered treatment/past 28 days |  | 0.0031 | 0.0074 | -0.0114 | 0.0176 | 0.68 |
| Main way took methamphetamine in the 28 days before entering treatment/past 28 days | Injection | 0.323 | 0.1831 | -0.0359 | 0.6819 | 0.08 |
|  | Others | – | – | – | – |  |
| The main form of methamphetamine used in the 28 days before entered treatment/past 28 days | Crystal meth or ice | 0.1234 | 0.1113 | -0.0947 | 0.3415 | 0.27 |
|  | Others | – | – | – | – |  |
| Methamphetamine Severity of Dependence Scale |  | 0.0151 | 0.017 | -0.0182 | 0.0483 | 0.37 |
| Polydrug use in the past 28 days |  | 0.0247 | 0.0406 | -0.0549 | 0.1043 | 0.54 |
| Major depression | No | -0.0996 | 0.1181 | -0.3311 | 0.1319 | 0.4 |
|  | Yes | – | – | – | – |  |
| Panic disorder or social phobia | No | 0.0655 | 0.128 | -0.1853 | 0.3163 | 0.61 |
|  | Yes | – | – | – | – |  |
| K10 Psychological Distress scale |  | 0.0149 | 0.0127 | -0.01 | 0.0398 | 0.24 |
| Jablensky psychosis screener | No | 0.0751 | 0.1396 | -0.1985 | 0.3487 | 0.59 |
|  | Yes | – | – | – | – |  |
| SF12 mental health component scale score |  | 0.0053 | 0.0079 | -0.0102 | 0.0208 | 0.50 |
| SF12 physical health component scale score |  | 0.0041 | 0.0067 | -0.009 | 0.0172 | 0.54 |

**Table S5. Drug treatment costs and total healthcare costs by participant characteristics**

|  | **Drug treatment costs** | | |  | **Total healthcare costs** | | |
| --- | --- | --- | --- | --- | --- | --- | --- |
|  | **N*** | **Mean (SD)** | **Median (IQR)** | **p-value**** | **Mean (SD)** | **Median (IQR)** | **p-value**** |
| Age |  |  |  | 0.08 |  |  | **0.05** |
| ≤31 | 171 | 11,284 (13,249) | 7767 (2491-13,272) |  | 19,154 (21,796) | 10,527 (5556-25,473) |  |
| >31 | 169 | 9787 (14,715) | 6340 (500-11,934) |  | 14,555 (17,575) | 9096 (4016-19,334) |  |
| Gender |  |  |  | **0.05** |  |  | 0.26 |
| Female | 103 | 8575 (10,867) | 4200 (2140-12,807) |  | 18,114 (19,656) | 12,943 (4,512-24,187) |  |
| Male | 237 | 11,355 (15,050) | 7767 (2520-12,645) |  | 16,340 (20,031) | 9309 (4269-20,406) |  |
| Education |  |  |  | 0.97 |  |  | 0.99 |
| Completed <10 years of school education | 101 | 10,623 (15,771) | 7137 (2140-12,294) |  | 16,228 (18,496) | 9846 (4379-22,776) |  |
| Completed ≥10 years of school education | 239 | 10,505 (13,201) | 7299 (2259-12,807) |  | 17,134 (20,517) | 9958 (4678-21,740) |  |
| Employment status |  |  |  | 0.34 |  |  | 0.59 |
| Unemployed | 271 | 10,383 (12,259) | 7767 (2223-12,807) |  | 16,492 (18,571) | 10,256 (4678-21,548) |  |
| Others | 69 | 11,165 (19,549) | 5020 (2140-11,172) |  | 18,330 (24,622) | 8327 (4210-24,093) |  |
| Fortnight legitimate income after-tax |  |  |  | 0.17 |  |  | 0.23 |
| <$400 | 179 | 12,340 (17,590) | 7767 (2925-15,964) |  | 18,304 (20,152) | 10,843 (4990-25,544) |  |
| ≥$400 | 161 | 9950 (12,583) | 6883 (2140-12,116) |  | 16,387 (19,846) | 9402 (4390-19,901) |  |
| Marital status |  |  |  | 0.28 |  |  | 0.73 |
| Others | 277 | 10,509 (12,443) | 7767 (2391-12,645) |  | 17,157 (19,963) | 9841 (4678-23,784) |  |
| Married/de-facto | 63 | 10,673 (19,448) | 4518 (2008-13,272) |  | 15,593 (19,782) | 10,871 (3661-19,221) |  |
| Accommodation |  |  |  | **0.04** |  |  | 0.56 |
| Stable accommodation | 214 | 11,179 (14,921) | 7767 (2520-12,681) |  | 17,412 (21,287) | 10,256 (5323-20,292) |  |
| Public housing | 49 | 8831 (13,518) | 3780 (0-9578) |  | 15,495 (17,301) | 8175 (3128-24,322) |  |
| Other accommodation | 77 | 9905 (11,511) | 7641 (1506-13,541) |  | 16,237 (17,592) | 10,366 (3930-25,346) |  |
| Prison history |  |  |  | **0.03** |  |  | **0.01** |
| No | 216 | 11,202 (14,560) | 7767 (2520-13,710) |  | 18,203 (20,554) | 10,676 (5556-25,174) |  |
| Yes | 124 | 9430 (12,975) | 5316 (0-11,760) |  | 14,622 (18,647) | 8876 (3050-18,307) |  |
| Ever injected a drug |  |  |  | 0.15 |  |  | 0.12 |
| No | 51 | 7509 (10,311) | 3989 (2368-7767) |  | 13,814 (18,284) | 7916 (3912-16,242) |  |
| Yes | 289 | 11,042 (14,468) | 7767 (2140-14,359) |  | 17,355 (20,145) | 10,352 (4678-23,784) |  |
| Number of days used methamphetamine in the 28 days before entered treatment/past 28 days |  |  |  | **<0.01** |  |  | **0.03** |
| 1-14 days | 16 | 8612 (11,279) | 5130 (753-10,080) |  | 14,824 (19,238) | 9106 (3904-19,278) |  |
| 15+ days | 324 | 12,182 (15,795) | 7767 (3159-15,243) |  | 18,585 (20,352) | 11,793 (5504-24,942) |  |
| Main way took methamphetamine in the 28 days before entering treatment/past 28 days |  |  |  | 0.15 |  |  | **0.03** |
| Others | 82 | 8017 (10,154) | 5040 (2391-7767) |  | 13,489 (17,076) | 8215 (4390-15,833) |  |
| Injection | 242 | 11,395 (14,998) | 7767 (2140-14,757) |  | 17,991 (20,679) | 10,942 (4658-24,942) |  |
| The main form of methamphetamine used in the 28 days before entered treatment/past 28 days |  |  |  | 0.73 |  |  | 0.88 |
| Others | 139 | 10,594 (16,015) | 6552 (2510-11,436) |  | 15,789 (19,103) | 9939 (5474-19,334) |  |
| Crystal meth or ice | 183 | 10,615 (12,325) | 7767 (2140-13,541) |  | 17,757 (20,573) | 9941 (3930-25,779) |  |
| Methamphetamine Severity of Dependence Scale |  |  |  | **<0.01** |  |  | **<0.01** |
| Low to moderate (0-9) | 149 | 8235 (12,710) | 4640 (1506-10,736) |  | 13,125 (15,382) | 8390 (3571-17,793) |  |
| High (10-15) | 175 | 13,023 (14,903) | 7767 (3218-15,966) |  | 20,861 (23,211) | 11,874 (5845-27,335) |  |
| Polydrug use in the past 28 days |  |  |  | 0.67 |  |  | 0.45 |
| 1-3 drugs | 180 | 10,255 (12,108) | 7767 (2140-13,272) |  | 16,069 (15,926) | 10,648 (4746-22,776) |  |
| 4+ drugs | 160 | 10,723 (15,466) | 6850 (2457-11,741) |  | 17,456 (22,787) | 9198 (4507-19,605) |  |
| Major depression |  |  |  | **0.02** |  |  | 0.94 |
| No | 161 | 11,617 (14,987) | 7767 (3035-14,786) |  | 17,011 (20,753) | 9888 (4510-23,679) |  |
| Yes | 176 | 9322 (12,719) | 5693 (546-11,504) |  | 16,691 (18,966) | 9998 (4493-21,536) |  |
| Panic disorder or social phobia |  |  |  | 0.22 |  |  | 0.20 |
| No | 180 | 10,911 (12,743) | 7575 (2457-15,052) |  | 15,723 (16,885) | 9309 (3574-22,563) |  |
| Yes | 160 | 9918 (15,912) | 6786 (2140-10,854) |  | 18,762 (24,085) | 10,688 (5930-21,453) |  |
| K10 Psychological Distress scale |  |  |  | **<0.05** |  |  | **<0.01** |
| No or mild mental disorder (10-24) | 211 | 6026 (7805) | 3260 (0-7767) |  | 1081 (10,643) | 5575 (2775-18,184) |  |
| Moderate mental disorder (25-29) | 129 | 8264 (10,464) | 4779 (1020-10,851) |  | 13,744 (16,806) | 8412 (3335-17,228) |  |
| Severe mental disorder (30+) |  | 12,474 (15,783) | 7767 (3159-14,992) |  | 19,693 (22,096) | 11,578 (6567-25,483) |  |
| Jablensky psychosis screener | 174 |  |  | 0.81 |  |  | 0.97 |
| No | 166 | 9723 (10,109) | 7752 (2457-14,757) |  | 14,506 (12,693) | 10,107 (4790-22,776) |  |
| Yes |  | 10799 (15,027) | 7186 (2140-12,030) |  | 17,612 (21,675) | 9941 (4390-21,740) |  |
| SF12 mental health component scale score | 81 |  |  | **0.03** |  |  | **<0.01** |
| Nil or mild (<40) | 259 | 7743 (10,511) | 3393 (0-11,085) |  | 11,079 (11,685) | 7902 (2785-17,274) |  |
| Moderate or severe stability (≥40) |  | 11,137 (14,578) | 7767 (2457-12,807) |  | 18,101 (21,071) | 10,400 (5323-24,187) |  |
| SF12 physical health component scale score | 171 |  |  | 0.61 |  |  | 0.80 |
| Nil or mild (<40) | 169 | 10,563 (12,982) | 7267 (2457-12,470) |  | 16,699 (19,677) | 9846 (4390-21,858) |  |
| Moderate or severe stability (≥40) |  | 10,474 (16,641) | 7452 (740-14,112) |  | 17,323 (20,662) | 10,113 (5083-22,666) |  |

IQR, interquartile range.

**Table S6. Breakdown of health services utilisation costs**

|  | **Overall** | | | | | | **Not in treatment** | | | | | | **Detoxification** | | | | | |
| --- | --- | --- | --- | --- | --- | --- | --- | --- | --- | --- | --- | --- | --- | --- | --- | --- | --- | --- |
|  | N | Mean | Std | Median | Q1 | Q3 | N | Mean | Std | Median | Q1 | Q3 | N | Mean | Std | Median | Q1 | Q3 |
| Health services utilisation | 323 | $6316 | $12,236 | $1643 | $550 | $5117 | 70 | $5100 | $6503 | $2229 | $579 | $6448 | 59 | $5245 | $8134 | $1147 | $448 | $5353 |
| General hospital | 324 | $1802 | $6602 | $0 | $0 | $0 | 70 | $1231 | $3190 | $0 | $0 | $870 | 59 | $1091 | $3021 | $0 | $0 | $0 |
| Emergency department | 324 | $435 | $811 | $0 | $0 | $652 | 70 | $397 | $751 | $0 | $0 | $686 | 59 | $409 | $634 | $0 | $0 | $865 |
| Ambulance | 324 | $288 | $675 | $0 | $0 | $0 | 70 | $255 | $525 | $0 | $0 | $777 | 59 | $356 | $583 | $0 | $0 | $777 |
| Psychiatric hospital | 324 | $2242 | $7901 | $0 | $0 | $0 | 70 | $679 | $3300 | $0 | $0 | $0 | 59 | $2455 | $7097 | $0 | $0 | $0 |
| Psychiatric | 324 | $488 | $1794 | $0 | $0 | $0 | 70 | $778 | $2638 | $0 | $0 | $275 | 59 | $135 | $517 | $0 | $0 | $0 |
| Psychologist/counsellor | 324 | $341 | $1247 | $0 | $0 | $187 | 70 | $844 | $2431 | $0 | $0 | $597 | 59 | $186 | $552 | $0 | $0 | $75 |
| Dentist | 324 | $194 | $323 | $0 | $0 | $179 | 70 | $243 | $409 | $0 | $0 | $358 | 59 | $139 | $215 | $0 | $0 | $179 |
| General practitioner | 323 | $585 | $718 | $373 | $149 | $895 | 70 | $674 | $838 | $373 | $149 | $895 | 59 | $474 | $645 | $298 | $75 | $671 |

|  | **Counselling** | | | | | | **Residential rehabilitation** | | | | | |
| --- | --- | --- | --- | --- | --- | --- | --- | --- | --- | --- | --- | --- |
|  | N | Mean | Std | Median | Q1 | Q3 | N | Mean | Std | Median | Q1 | Q3 |
| Health services utilisation | 29 | $13,632 | $24,830 | $1492 | $552 | $16,895 | 165 | $5929 | $11,698 | $1524 | $552 | $4084 |
| General hospital | 29 | $4148 | $12,568 | $0 | $0 | $1071 | 166 | $1886 | $7055 | $0 | $0 | $0 |
| Emergency department | 29 | $589 | $974 | $0 | $0 | $865 | 166 | $433 | $862 | $0 | $0 | $579 |
| Ambulance | 29 | $322 | $918 | $0 | $0 | $0 | 166 | $272 | $715 | $0 | $0 | $0 |
| Psychiatric hospital | 29 | $6399 | $17,595 | $0 | $0 | $0 | 166 | $2099 | $6580 | $0 | $0 | $0 |
| Psychiatric | 29 | $1119 | $3186 | $0 | $0 | $275 | 166 | $381 | $1188 | $0 | $0 | $0 |
| Psychologist/counsellor | 29 | $188 | $411 | $0 | $0 | $224 | 166 | $210 | $546 | $0 | $0 | $75 |
| dentist | 29 | $203 | $312 | $0 | $0 | $358 | 165 | $192 | $314 | $0 | $0 | $358 |
| General practitioner | 29 | $664 | $792 | $448 | $224 | $895 | 165 | $573 | $674 | $298 | $149 | $895 |

Note: We based on the information provided in case report forms to do the costing. If the information was not provided, we used published average costs in the Government reports. General hospital cost: $8594 -Overnight; Non-overnight general hospital: $680.44; emergency department costs: $956 -Overnight; non-overnight emergency department costs: $443; Ambulance costs: $777.4; Psychiatric hospital: $18,102 -Overnight; Non-overnight psychiatric hospital: $11,331; Psychology: $275 per session, or $74.6 per hour; Dentist costs: $178.75; General practitioner: $74.6.

**Table S7. Australian Refined Diagnosis Related Group (AR-DRG) used, frequencies, case weights and costs in the study**

| **Code DRG** | **Description** | **Freq** | **Case weight** | **Costs** |
| --- | --- | --- | --- | --- |
| X62A | Poisoning/toxic effects of drugs and other substances, major complexity, same day | 2 | 0.1532 | $1491 |
| J64A | Cellulitis, major complexity | 1 | 1.3322 | $6482 |
| Y02A | Skin grafts, major complexity | 1 | 8.1483 | $39,650 |
| X60A | Injuries, major complexity | 6 | 0.1802 | $5261 |
| I74A | Injuries to forearm, wrist, hand, and foot, major complexity | 1 | 0.7718 | $3756 |
| W04B | Multiple significant traumas with other general interventions, minor complexity | 2 | 4.35 | $42,334 |
| V61B | Drug intoxication and withdrawal, minor complexity | 1 | 0.81 | $3941 |
| X60B | Injuries, minor complexity | 4 | 0.2044 | $3978 |
| I30Z | Hand procedures | 1 | 0.83 | $4039 |
| T61B | Postoperative infections, minor complexity | 1 | 0.6248 | $3040 |
| X60A | Injuries, major complexity | 3 | 1.0245 | $14,956 |
| O60C | Vaginal delivery, minor complexity | 1 | 0.8454 | $4114 |
| B80B | Other head injuries, minor complexity | 1 | 0.1422 | $692 |
| D12A | Other ear, nose, mouth, and throat procedures a major complexity | 1 | 0.7389 | $3595 |
| I16Z | Other shoulder procedures | 1 | 0.7389 | $3595 |
| B76A | Seizures, major complexity | 2 | 0.2215 | $2156 |
| V61A | Drug intoxication and withdrawal, major complexity | 1 | 2.0249 | $9853 |
| G07B | Appendicectomy, minor complexity | 2 | 1.2016 | $11,694 |
| K60B | Diabetes, minor complexity | 1 | 0.7764 | $3778 |
| I16Z | Other shoulder procedures | 2 | 1.551 | $15,094 |
| U63B | Major affective disorders, minor complexity | 2 | 3.4084 | $33,171 |
| H07B | Open cholecystectomy, intermediate complexity | 1 | 3.7865 | $18,425 |
| G67B | Oesophagitis and gastroenteritis, minor complexity | 1 | 0.2202 | $1071 |
| I28C | Other musculoskeletal procedures, minor complexity | 5 | 0.555 | $13,503 |
| M04Z | Testes procedures | 2 | 0.8266 | $8044 |
| Z01B | Other contacts with health services with general interventions, minor complexity | 4 | 0.6858 | $13,348 |
| B06A | Procedures for cerebral palsy, muscular dystrophy and neuropathy, major comp | 2 | 6.4464 | $62,736 |
| N08Z | Endoscopic and laparoscopic procedures, female reproductive system | 1 | 1.3746 | $6689 |
| O01A | Cesarean delivery, major complexity | 3 | 3.1885 | $46,546 |
| U65A | Anxiety disorders, major complexity | 1 | 2.5247 | $12,285 |
| J64B | Cellulitis, minor complexity | 1 | 0.6565 | $3195 |
| I19B | Other elbow and forearm procedures, minor complexity | 2 | 1.5454 | $15,040 |
| J10A | Plastic general interventions for skin, subcutaneous tissue and breast disorders, major comp | 2 | 2.0694 | $20,139 |
| U62A | Paranoia and acute psychotic disorders, major complexity | 1 | 5.9315 | $28,863 |
| N04A | Hysterectomy for non-malignancy, major complexity | 1 | 2.922 | $14,218 |
| U62B | Paranoia and acute psychotic disorders, minor complexity | 1 | 3.0138 | $3018 |
| Z63B | Other follow-up after surgery or medical care, minor complexity | 1 | 0.6203 | $3018 |
| Z61B | Signs and symptoms, minor complexity | 4 | 0.2265 | $4409 |
| X62B | Poisoning/toxic effects of drugs and other substances, minor complexity | 3 | 0.2533 | $3698 |
| Z61B | Signs and symptoms, minor complexity | 1 | 0.4995 | $2431 |
| Z61A | Signs and symptoms, major complexity | 1 | 1.2735 | $6197 |
| T60C | Septicaemia, minor complexity | 1 | 0.9682 | $4711 |
| B77B | Headaches, minor complexity | 1 | 0.1788 | $870 |
| B76A | Seizures, major complexity | 3 | 1.2741 | $18,599 |
| J11B | Other skin, subcutaneous tissue and breast procedures, minor complexity | 1 | 0.4437 | $2159 |
| O63B | Abortion without general interventions, minor complexity | 1 | 0.2099 | $1021 |
| Z64B | Other factors influencing health status, minor complexity | 3 | 0.2013 | $2939 |
| H63C | Other disorders of the liver, minor complexity | 1 | 0.2482 | $1208 |
| E62B | Respiratory infections and inflammations, minor complexity | 1 | 0.7332 | $3568 |
| O66C | Antenatal and other obstetric admissions, minor complexity | 1 | 0.1305 | $635 |
| O02A | Vaginal delivery with general interventions, major complexity | 1 | 2.3555 | $11,462 |
| I03B | Hip replacement for trauma, minor complexity | 1 | 3.9036 | $18,995 |
| G47C | Gastroscopy, minor complexity | 1 | 0.283 | $1377 |
| F76A | Arrhythmia, cardiac arrest, and conduction disorders, major complexity | 1 | 1.0982 | $5344 |
| J09Z | Perianal and pilonidal procedures | 1 | 0.6978 | $3395 |
| D63A | Otitis media and upper respiratory infections, major complexity | 1 | 0.8211 | $3995 |
| Others |  | 21 | 680.44 | $14,289 |
| Total |  |  |  | $609,760 |

Note: 21 cases were not assigned AR-DRG codes, we used average hospitalisation costs.

# **Table S8. Unit cost of drug treatment by treatment modality**

| **Treatment modality** | **Cost per episode per person (year 2019*)** | **Source** |
| --- | --- | --- |
| **Residential rehabilitation** | $428 per day | [34] |
| **Counselling** | $2136 per episode | [34] |
| **Inpatient detox** | $7756 per episode | [34] |
| **Outpatient detox** | $5123 per episode | [34] |

*Costs were adjusted to 2019 currency by using consumer price index [25].

**Table S9. CHEERS Checklist**

|  | **Item** | **Guidance for reporting** | **Reported in section** |
| --- | --- | --- | --- |
| **TITLE** | | |  |
| Title | 1 | Identify the study as an economic evaluation and specify the interventions being compared. | Page 1 |
| **ABSTRACT** | | |  |
| Abstract | 2 | Provide a structured summary that highlights context, key methods, results and alternative analyses. | Page 2 |
| **INTRODUCTION** | | |  |
| Background and objectives | 3 | Give the context for the study, the study question, and its practical relevance for decision-making in policy or practice. | Page 4 |
| **METHODS** | | |  |
| Health economic analysis plan | 4 | Indicate whether a health economic analysis plan was developed and where available. | Page 6, 7 |
| Study population | 5 | Describe characteristics of the study population (such as age range, demographics, socioeconomic, or clinical characteristics). | Page 6 |
| Setting and location | 6 | Provide relevant contextual information that may influence findings. | Page 6 |
| Comparators | 7 | Describe the interventions or strategies being compared and why chosen. | Page 6, 7 |
| Perspective | 8 | State the perspective(s) adopted by the study and why chosen. | Page 6 |
| Time horizon | 9 | State the time horizon for the study and why appropriate. | NA |
| Discount rate | 10 | Report the discount rate(s) and reason chosen. | NA |
| Selection of outcomes | 11 | Describe what outcomes were used as the measure(s) of benefit(s) and harm(s). | Page 7, 8 |
| Measurement of outcomes | 12 | Describe how outcomes used to capture benefit(s) and harm(s) were measured. | Page 6, 7, 8 |
| Valuation of outcomes | 13 | Describe the population and methods used to measure and value outcomes. | Page 6, 7, 8 |
| Measurement and valuation of resources and costs | 14 | Describe how costs were valued. | Page 6, 7, 8 |
| Currency, price date, and conversion | 15 | Report the dates of the estimated resource quantities and unit costs, plus the currency and year of conversion. | Page 6, 7, 8 |
| Rationale and description of the model | 16 | If modelling is used, describe in detail why used. Report if the model is publicly available and where it can be accessed. | NA |
| Analytics and assumptions | 17 | Describe any methods for analysing or statistically transforming data, any extrapolation methods, and approaches for validating any model used. | NA |
| Characterising heterogeneity | 18 | Describe any methods used for estimating how the results of the study vary for sub-groups. | Page 8 |
| Characterising distributional effects | 19 | Describe how impacts are distributed across different individuals or adjustments made to reflect priority populations. | NA |
| Characterising uncertainty | 20 | Describe methods to characterize any sources of uncertainty in the analysis. | NA |
| Approach to engagement with patients and others affected by the study | 21 | Describe any approaches to engage patients or service recipients, the general public, communities, or stakeholders (e.g., clinicians or payers) in the design of the study. | NA |
| **RESULTS** | | |  |
| Study parameters | 22 | Report all analytic inputs (e.g., values, ranges, references) including uncertainty or distributional assumptions. | Page 10, 11 |
| Summary of main results | 23 | Report the mean values for the main categories of costs and outcomes of interest and summarize them in the most appropriate overall measure. | Page 10, 11 |
| Effect of uncertainty | 24 | Describe how uncertainty about analytic judgments, inputs, or projections affects findings. Report the effect of the choice of discount rate and time horizon, if applicable. | NA |
| Effect of engagement with patients and others affected by the study | 25 | Report on any difference between patient/service recipient, general public, community, or stakeholder involvement made to the approach or findings of the study | NA |
| **DISCUSSION** | | |  |
| Study findings, limitations, generalisability, and current knowledge | 26 | Report key findings, limitations, ethical or equity considerations not captured, and how these could impact patients, policy, or practice. | Page 12, 13 |
|  | | | |
| Source of funding | 27 | Describe how the study was funded and any role of the funder in the identification, design, conduct, and reporting of the analysis | Page 15 |
| Conflicts of interest | 28 | Report the author's conflicts of interest according to the journal or International Committee of Medical Journal Editors requirements. | Page 15 |

# **S10. Treatment for methamphetamine**

**PART A Index treatment**

***Treatment duration and completion***

E 1. When you were last interviewed for this study (about 3 months ago) you were :

1. in out-patient counselling at ( ) 1
2. in a residential rehab at ( ) 2
3. in a detox unit at ( ).......................... 3 **Go to E3**
4. doing outpatient detox through ( ). 3 **Go to E3**
5. not in treatment............................................................................. 4 **Go to Part B E11**

E 2. Are you still in this treatment (exclude follow-up care if index tx was residential)?

No 0

Yes .......................... 1 **Go to E7**

E 3. When did you leave this treatment (include only the in-patient component if index treatment was residential)?

Day / Month / Year

E 4. Did you remain in this treatment continuously or did you take a break from treatment?

1. Stayed in treatment 1
2. Had time out 0 (Record time out of treatment: days)

E 5. (a) Did you complete the treatment? (include only in-patent component if index treatment was residential)

No 0

Yes .......................... 1 **Skip to E 6**

(b) What was the reason you left this treatment? Did you:

Transfer to another service 2

Left without notice 3

Left against advice 4

Involuntary 5

Moved out of area 6

Sanctioned by Drug Court 7

Other imprisonment 8

Other.............................................9 Specify:

Comments:

E 6. (a) If treatment was inpatient service, ask:

Did this treatment involve an outpatient follow-up program? No............................ 0 **Skip to E 7**

Yes 1

1. Have you completed the outpatient follow-up care?

No............................ 0 **Skip to E 7**

Yes 1

1. Date completed outpatient care:

Day / Month / Year

***The type of treatment provided***

E 7. What was the main drug that you received help for during this treatment? (Prompt: Was it methamphetamine, or was it another drug?)

Methamphetamine 1

Heroin 2

Cocaine 3

Cannabis 4

Alcohol 5

Other .................................... 6 Specify

E 8. What other drugs did you receive help for on this occasion (can mark more than one)?

Methamphetamine 1

Heroin 2

Cocaine 3

Cannabis 4

Alcohol 5

Other .................................... 6 Specify

E 9. (a) Were you given any medication as part of this treatment?

No............................ 0 **Skip to E 10**

Yes 1

1. What medications were you given (can mark more than one)?

Specify brand name(s) Benzodiazepines................... 1

Antidepressants ................... 2

Anti-psychotics .................... 3

Barbiturates .......................... 4

Other..................................... 5

*Record in the table below:*

E 10. A. i) How many individual counselling sessions did you do while you were in this treatment?

ii) How many of these individual counselling sessions involved discussing your drug use (as opposed to other lifestyle issues)?

- 1. i) How many group counselling sessions did you do while you were in this treatment?
     1. How many of these group counselling sessions involved discussing your drug use (as opposed to other lifestyle issues)?

*If the participant is involved in outpatient/follow-up care, ask:*

- 1. i) How many individual counselling sessions did you do while you were in outpatient follow-up care?
     1. How many of these individual counselling sessions involved discussing your drug use (as opposed to other lifestyle issues)?
  2. i) How many group counselling sessions did you do while you were in outpatient follow-up care?
     1. How many of these group counselling sessions involved discussing your drug use (as opposed to other lifestyle issues)?

| INPATIENT FOLLOW-UP CARE | | | | | |
| --- | --- | --- | --- | --- | --- |
| i)Sessions ii)Drug use i)Sessions ii)Drug use | | | | | |
| A.Individual |  |  | C.Individual |  |  |
| B.Group |  |  | D.Group |  |  |

**PART B Other treatment episodes**

**Identification of other treatment episodes**

E 11. Have you **started** treatment for your drug or alcohol use [at any **other** drug treatment services] since we last interviewed you?

No............................ 0 **Skip to Section F**

Yes 1

E 12. How many other times have you started treatment for your drug or alcohol use since we last interviewed you (count detox as a separate TX episode, even if done to enter counselling or residential rehab)?

**Second treatment occasion**

You mentioned that you have been to treatment x times since we first interviewed you. E 13. What type of treatment did you receive on this occasion?

Inpatient withdrawal (with or without medication) 1

Outpatient withdrawal (with or without medication) 2

Counselling^1^ (not as a part of other treatment) 3

Residential Rehabilitation (e.g., Therapeutic Community):

RR with detox 4

RR without detox 5

Other: Specify 6

1. ***Interviewer:*** *Name of the treatment centre from which the client received treatment:*

E 14. (a) Were you required to attend this drug treatment as the result of a drug court order?

No 0

Yes 1 **Skip to E 15**

1. Were you required to attend this treatment for any other legal reason?

No 0

Yes: Specify 1

***Treatment duration and completion***

E 15. When did you start this treatment?

Day / Month / Year

E 16. Are you still in this treatment (exclude follow-up care if index tx was residential)?

No 0

Yes .......................... 1 **Go to E 19 (Treatment provided)**

E 17. When did you leave this treatment?

Day / Month / Year

^1^ Counselling includes both face-to-face and group counselling that is EITHER (a) provided by a certified counselling practitioner, including registered psychologists and psychiatrists, OR (b) counselling provided within the context of a drug and alcohol treatment service. Counselling, case management or other supportive services provided outside this context should be coded as ëotherí (e.g., outreach, general practitioner)

E 18. Did you remain in this treatment continuously or did you take a break from treatment?

- 1. Stayed in treatment 1
  2. Had time out 0 (Record time out of treatment: days)

***The type of treatment provided***

E 19. What was the main drug that you received help for during this treatment? (Prompt: Was it methamphetamine, or was it another drug?)

Methamphetamine 1

Heroin 2

Cocaine 3

Cannabis 4

Alcohol 5

Other .................................... 6 Specify

E 20. What other drugs did you receive help for on this occasion (can mark more than one)?

Methamphetamine 1

Heroin 2

Cocaine 3

Cannabis 4

Alcohol 5

Other .................................... 6 Specify

E 21. Record the number of counselling sessions in the table below.

1. i) How many individual counselling sessions did you do while you were in this treatment?
   1. How many of these individual counselling sessions involved discussing your drug use (as opposed to other lifestyle issues)?
2. i) How many group counselling sessions did you do while you were in this treatment?
   1. How many of these group counselling sessions involved discussing your drug use (as opposed to other lifestyle issues)?

*If the participant is involved in outpatient/follow-up care, ask:*

1. i) How many individual counselling sessions did you do while you were in outpatient follow-up care?
   1. How many of these individual counselling sessions involved discussing your drug use (as opposed to other lifestyle issues)?
2. i) How many group counselling sessions did you do while you were in outpatient follow-up care?
   1. How many of these group counselling sessions involved discussing your drug use (as opposed to other lifestyle issues)?

| INPATIENT FOLLOW-UP CARE | | | | | |
| --- | --- | --- | --- | --- | --- |
| i)Sessions ii)Drug use i)Sessions ii)Drug use | | | | | |
| A.Individual |  |  | C.Individual |  |  |
| B.Group |  |  | D.Group |  |  |

**Third treatment occasion**

You mentioned that you have been to treatment x times since we first interviewed you.

E 22. What type of treatment did you receive on this occasion?

Inpatient withdrawal (with or without medication) 1

Outpatient withdrawal (with or without medication) 2

Counselling^2^ (not as a part of other treatment) 3

Residential Rehabilitation (e.g., Therapeutic Community):

RR with detox 4

RR without detox 5

Other: Specify 6

1. ***Interviewer:*** *Name of the treatment centre from which the client received treatment:*

E 23. (a) Were you required to attend this drug treatment as the result of a drug court order?

No 0

Yes 1 **Skip to E 24**

1. Were you required to attend this treatment for any other legal reason?

No 0

Yes: Specify 1

***Treatment duration and completion***

E 24. When did you start this treatment?

Day / Month / Year

E 25. Are you still in this treatment (exclude follow-up care if index tx was residential)?

No 0

Yes .......................... 1 **Go to E 28 (Treatment provided)**

E 26. When did you leave this treatment?

Day / Month / Year

^2^ Counselling includes both face-to-face and group counselling that is EITHER (a) provided by a certified counselling practitioner, including registered psychologists and psychiatrists, OR (b) counselling provided within the context of a drug and alcohol treatment service. Counselling, case management or other supportive services provided outside this context should be coded as ëotherí (e.g., outreach, general practitioner)

E 27. Did you remain in this treatment continuously or did you take a break from treatment?

- 1. Stayed in treatment 1
  2. Had time out 0 (Record time out of treatment: days)

***The type of treatment provided***

E 28. What was the main drug that you received help for during this treatment? (Prompt: Was it methamphetamine, or was it another drug?)

Methamphetamine 1

Heroin 2

Cocaine 3

Cannabis 4

Alcohol 5

Other .................................... 6 Specify

E 29. What other drugs did you receive help for on this occasion (can mark more than one)?

Methamphetamine 1

Heroin 2

Cocaine 3

Cannabis 4

Alcohol 5

Other .................................... 6 Specify

*Record in the table below:*

E 30. Record the number of counselling sessions in the table below.

1. i) How many individual counselling sessions did you do while you were in this treatment?
   1. How many of these individual counselling sessions involved discussing your drug use (as opposed to other lifestyle issues)?
2. i) How many group counselling sessions did you do while you were in this treatment?
   1. How many of these group counselling sessions involved discussing your drug use (as opposed to other lifestyle issues)?

*If the participant is involved in outpatient/follow-up care, ask:*

1. i) How many individual counselling sessions did you do while you were in outpatient follow-up care?

ii) How many of these individual counselling sessions involved discussing your drug use (as opposed to other lifestyle issues)?

1. i) How many group counselling sessions did you do while you were in outpatient follow-up care?

ii) How many of these group counselling sessions involved discussing your drug use (as opposed to other lifestyle issues)?

| INPATIENT FOLLOW-UP CARE | | | | | |
| --- | --- | --- | --- | --- | --- |
| i)Sessions ii)Drug use i)Sessions ii)Drug use | | | | | |
| A.Individual |  |  | C.Individual |  |  |
| B.Group |  |  | D.Group |  |  |

**Fourth treatment occasion**

You mentioned that you have been to treatment x times since we first interviewed you.

E 31. What type of treatment did you receive on this occasion?

Inpatient withdrawal (with or without medication) 1

Outpatient withdrawal (with or without medication) 2

Counselling^3^ (not as a part of other treatment) 3

Residential Rehabilitation (e.g., Therapeutic Community):

RR with detox 4

RR without detox 5

Other: Specify 6

1. ***Interviewer:*** *Name of the treatment centre from which the client received treatment:*

E 32. (a) Were you required to attend this drug treatment as the result of a drug court order?

No 0

Yes 1 **Skip to E 33**

1. Were you required to attend this treatment for any other legal reason?

No 0

Yes: Specify 1

***Treatment duration and completion***

E 33. When did you start this treatment?

Day / Month / Year

E 34. Are you still in this treatment (exclude follow-up care if index tx was residential)?

No 0

Yes .......................... 1 **Go to E 37** (Treatment provided)

E 35. When did you leave this treatment?

Day / Month / Year

^3^ Counselling includes both face-to-face and group counselling that is EITHER (a) provided by a certified counselling practitioner, including registered psychologists and psychiatrists, OR (b) counselling provided within the context of a drug and alcohol treatment service. Counselling, case management or other supportive services provided outside this context should be coded as ëotherí (e.g., outreach, general practitioner)

E 36. Did you remain in this treatment continuously or did you take a break from treatment?

- 1. Stayed in treatment 1
  2. Had time out 0 (Record time out of treatment: days)

***The type of treatment provided***

E 37. What was the main drug that you received help for during this treatment? (Prompt: Was it methamphetamine, or was it another drug?)

Methamphetamine 1

Heroin 2

Cocaine 3

Cannabis 4

Alcohol 5

Other .................................... 6 Specify

E 38. What other drugs did you receive help for on this occasion (can mark more than one)?

Methamphetamine 1

Heroin 2

Cocaine 3

Cannabis 4

Alcohol 5

Other .................................... 6 Specify

E 39. *Record the number of counselling sessions in the table below.*

1. i) How many individual counselling sessions did you do while you were in this treatment?
   1. How many of these individual counselling sessions involved discussing your drug use (as opposed to other lifestyle issues)?
2. i) How many group counselling sessions did you do while you were in this treatment?
   1. How many of these group counselling sessions involved discussing your drug use (as opposed to other lifestyle issues)?

*If the participant involved in outpatient/follow-up care, ask:*

C i) How many individual counselling sessions did you do while you were in outpatient follow-up care?

ii) How many of these individual counselling sessions involved discussing your drug use (as opposed to other lifestyle issues)?

D. i) How many group counselling sessions did you do while you were in outpatient follow-up care?

ii) How many of these group counselling sessions involved discussing your drug use (as opposed to other lifestyle issues)?

| INPATIENT FOLLOW-UP CARE | | | | | |
| --- | --- | --- | --- | --- | --- |
| i)Sessions ii)Drug use i)Sessions ii)Drug use | | | | | |
| A.Individual |  |  | C.Individual |  |  |
| B.Group |  |  | D.Group |  |  |

**S11. Health services utilisation**

O1 How many times have you been admitted to a general hospital in the past month? (*do not include psychiatric hospital attendances*)

O1.2. How many of these times were you admitted for at least one night?

O1.3.(For that admission/ Over those (O1.2) admissions) how many nights in total did you stay in a general hospital?

Reason for admission/s:

O2 How many times have you attended an emergency department in the past month?

O2.2. How many of these times did you stay in the emergency department for at least one night?

O2.3.(For that admission/ Over those (O2.2) admissions,) how many nights in total did you stay in an emergency department?

Reason for admission/s:

O3.1. How many times have you received help from an ambulance in the past month?

O3.2. How many of these times did you get taken to hospital by the ambulance?

O4. How many times have you been admitted to a psychiatric hospital in the past month?

O4.2. How many of these times were you admitted for at least one night to a psychiatric hospital?

O4.3. (For that admission/ Over those (O4.2) admissions,) how many nights in total did you stay in a psychiatric hospital?

O5. How many times have you been to see a psychiatrist in the past month (other than during an admission to the hospital or a drug tx)?

O6 How many times have you been to see a psychologist or counsellor in the past month (other than during an admission to the hospital or a drug tx)?

O7 How many times have you been to see a dentist in the past month?

O8a How many times have you been to see a general practitioner in the past month?

O8b How many times have you received help from any other health service in the past month? Type of health service:

O9 **Prescription medications**

O9.1. How many prescriptions did you get from any medical professional in the past month? (exclude repeats)

O9.2. How many times in the past month did you fill a script for the following drugs?
